# O157:H7 Shiga Toxin-Producing *Escherichia coli* Strains Associated with Sporadic Cases of Diarrhea in São Paulo, Brazil

**DOI:** 10.3201/eid0804.010490

**Published:** 2002-04

**Authors:** Kinue Irino, Tânia M. Ibelli Vaz, Maria A.M.F. Kato, Zita V.F. Naves, Raquel Russo Lara, Maria Elza Carvalho Marco, Marilu M.M. Rocha, Tânia P. Moreira, Tânia A. T. Gomes, Beatriz E.C. Guth

**Affiliations:** * Instituto Adolfo Lutz, São Paulo, Brazil; † Laboratório de Análises Clínicas Dr João Antônio Vozza, Campinas, São Paulo, Brazil; ‡ Instituto Adolfo Lutz, Campinas, São Paulo, Brazil; § Universidade Federal de São Paulo, Escola Paulista de Medicina, São Paulo, Brazil

**To the Editor:** Shiga toxin-producing *Escherichia coli* (STEC) strains are associated with a spectrum of diseases ranging from mild to severe bloody diarrhea and complications such as hemolytic uremic syndrome (HUS) and thrombotic thrombocytopenic purpura (*1*). Since STEC was linked with hemorrhagic colitis in 1982 (*2*), strains—particularly serotype O157:H7—have been associated with numerous outbreaks and many sporadic cases of infections worldwide. STEC is now a major cause of foodborne disease, primarily in the United States, Canada, Japan, and Europe (*1*,*3*). Although most sporadic cases and outbreaks have been reported from developed countries, human infections associated with STEC strains have also been described in Latin American countries, including Argentina and Chile (*3*). In Brazil, STEC infections have been related to sporadic cases of nonbloody diarrhea caused by non-O157 strains (*4*,*5*); serotype O157:H7 has not been previously isolated from human infections in our country.

We report the characterization of three O157:H7 strains isolated in São Paulo State, Brazil. The first strain was identified among a laboratory collection of 2,573 *E. coli* strains that were retrospectively analysed and isolated from patients with diarrhea in São Paulo State, from 1976 through 1999, at the Central Laboratory of Instituto Adolfo Lutz (IAL). This strain was isolated in 1990 from an 18-year-old patient with diarrheal disease who had AIDS. The two other O157 strains were recently isolated from a 4-year-old girl with bloody diarrhea and from an adult with severe diarrhea. Both patients were admitted to the same hospital at Campinas, São Paulo State, in June and July 2001, respectively. The strains, isolated by routine diagnostic procedures on MacConkey agar plates, were presumptively identified as *E. coli* O157 by standard methods with specific O157 antiserum. These last two strains were confirmed as sorbitol-negative *E. coli* O157 at the IAL Regional Laboratory at Campinas and were sent to the IAL Central Laboratory for further characterization.

The three O157 *E. coli* strains underwent biochemical identification and serotyping by standard methods. Enterohemolysin production was determined according to Beutin et al. (*6*). Polymerase chain reaction assays were used to detect the *stx1* and *stx2* genes (*7*), and colony hybridization assays with specific DNA probes for *stx1 , stx2* and *eae* genes were performed as described (*8*). Cytotoxicity to Vero cells was assayed as described (*4*). The strains were characterized as sorbitol-negative O157:H7, containing the *stx2* and *eae* sequences. The enterohemolytic phenotype and production of Stx were also observed in all isolates.

To our knowledge, these O157:H7 STEC strains are the first to be associated with human diseases in Brazil. Cultivation of stool specimens in sorbitol MacConkey agar is strongly recommended for screening O157 strains, and, indeed, all three strains were isolated from MacConkey agar plates. Laboratories should attempt to examine stool specimens from all patients (children and adults) with HUS, severe diarrhea (nonbloody and bloody stools) requiring hospitalization, or both, as well as from patients reporting a history of bloody diarrhea.

Despite the importance of O157:H7 serotype in causing life-threatening complications such as HUS and the isolation of this serotype from clinical specimens in São Paulo State, the relatively low prevalence of this serotype in healthy dairy and beef cattle in Brazil (*9*), as well as the occurrence of other non-O157 STEC strains associated with human infections (*4*,*5*,*10*), suggest that *E. coli* O157:H7 may be not as frequent as non-O157 STEC strains in our country.
