# O157:H7 Shiga Toxin-Producing *Escherichia coli* Strains Associated with Sporadic Cases of Diarrhea in São Paulo, Brazil

**DOI:** 10.3201/eid0805.020504

**Published:** 2002-05

## Correction, Vol. 8, No. 4

In the Letter to the Editor “O157:H7 Shiga Toxin-Producing Escherichia coli Strains Associated with Sporadic Cases of Diarrhea in São Paulo, Brazil,” by Kinue Irino et al., reference no. 1 was inadvertently omitted. That reference is

Griffin P, Tauxe RV. The epidemiology of infections caused by Escherichia coli O157:H7, other enterohemorrhagic E. coli, and the associated hemolytic uremic syndrome. Epidemiol Rev 1991;13:60-98. 

References in the text should be renumbered accordingly. The corrected version appears online at http://www.cdc.gov/ncidod/EID/vol8no4/01-0490.htm. We regret any confusion this error may have caused.

